# The Value of CT for Disease Detection and Prognosis Determination in Combined Pulmonary Fibrosis and Emphysema (CPFE)

**DOI:** 10.1371/journal.pone.0107476

**Published:** 2014-09-09

**Authors:** Seung Hee Choi, Ho Yun Lee, Kyung Soo Lee, Man Pyo Chung, O. Jung Kwon, Joungho Han, Namkug Kim, Joon Beom Seo

**Affiliations:** 1 Department of Radiology and Center for Imaging Science, Samsung Medical Center, Sungkyunkwan University School of Medicine, Seoul, Korea; 2 The Division of Respiratory and Critical Medicine of the Department of Internal Medicine, Samsung Medical Center, Sungkyunkwan University School of Medicine, Seoul, Korea; 3 The Department of Pathology, Samsung Medical Center, Sungkyunkwan University School of Medicine, Seoul, Korea; 4 Department of Radiology and Research Institute of Radiology, Asan Medical Center, University of Ulsan College of Medicine, Seoul, Korea; Clinica Universidad de Navarra, Spain

## Abstract

**Background and Purpose:**

Several imaging-based indices were constructed quantitatively using the emphysema index (EI) and fibrosis score (FS) on high-resolution computed tomography (HRCT). We evaluated the ability of these indices to predict mortality compared to physiologic results. Additionally, prognostic predictive factors were compared among subgroups with biopsy-proven fibrotic idiopathic interstitial pneumonia (IIP) (biopsy-proven CPFE) and in a separate cohort with subclinical CPFE.

**Materials and Methods:**

Three chest radiologists independently determined FS. EI was automatically quantified. PFTs, smoking history, and composite physiologic index (CPI) were reviewed. Predictors of time to death were determined based on clinico-physiologic factors and CT-based CPFE indices.

**Results:**

The prevalence of biopsy-proven CPFE was 26% (66/254), with an EI of 9.1±7.1 and a FS of 19.3±14.2. In patients with CPFE, median survival and 5-year survival rates were 6.0 years and 34.8%, respectively, whereas those in fibrotic IIP without emphysema were 10.0 years and 60.9% (*p* = 0.013). However, the extent of fibrosis did not differ significantly between the two cohorts. In subclinical CPFE, prevalence was 0.04% (93/20,372), EI was 11.3±10.4, and FS was 9.1±7.1. FVC and a fibrosis-weighted CT index were independent predictors of survival in the biopsy-proven CPFE cohort, whereas only the fibrosis-weighted CT index was a significant prognostic factor in the subclinical CPFE cohort.

**Conclusions:**

Recognition and stratification using CT quantification can be utilized as a prognostic predictor. Prognostic factors vary according to fibrosis severity and among cohorts of patients with biopsy-proven and subclinical CPFE.

## Introduction

In idiopathic pulmonary fibrosis (IPF), there is a dire need for accurate noninvasive measures of disease severity [1]. Variables including pulmonary function tests (PFT), extent of desaturation during a 6-min walk test, changes in forced vital capacity (FVC) and diffusion capacity for carbon monoxide (DLco) have been shown to have prognostic value [2]–[5]. Among routine indices, the DLco has the strongest correlation with the morphologic extent of disease, both histologically and on high-resolution computed tomography (HRCT). However, the quantification of disease severity using PFTs is often confounded by the concurrent presence of emphysema, resulting in spurious preservation of lung volumes and devastating depression of gas transfer [6], [7]. Wiggins *et al.* [8] demonstrated that these atypical physiologic and radiological features can be explained by co-existent IPF and emphysema, and that HRCT is valuable for assessing these patients. Furthermore, the overall extent of fibrosis itself seen on HRCT is an important independent predictor of mortality in patients with IPF [9], [10].

The composite physiologic index (CPI) was developed to improve upon previous prognostic measures in IPF by adjusting for emphysema and incorporating multiple components of pulmonary function, such as forced expiratory volume in 1 second (FEV_1_), FVC and DL_CO_ [1]. CPI score at diagnosis predicted mortality more accurately than individual PFT alone in patients with concomitant IPF and emphysema [1], [11]. However, according to a recent study that evaluated whether emphysema affected CPI or other individual measures of PFT in predicting patient mortality [11], individual measures of pulmonary function worked differently in predicting patient mortality based on the extent of emphysema. In other words, the initial hypothesis was that CPI would be the strongest predictor of mortality in patients with combined pulmonary fibrosis and emphysema (CPFE). However, the data indicated that changes in FEV1 were the best surrogate for predicting mortality in IPF patients as the extent of emphysema increased in fibrotic lungs. These results emphasized the importance of identifying and estimating the severity of emphysema for the optimization of patient outcomes [11].

In the present study, several imaging-based indices were constructed quantitatively using the emphysema index (EI) and the fibrosis score (FS) on HRCT. We evaluated the ability of these indices to predict mortality and compared their performance with those of previous physiologic factors. Additionally, prognostic predictive factors were compared among subgroups with biopsy-proven fibrotic idiopathic interstitial pneumonia (IIP) and in a separate earlier cohort.

## Materials and Methods

Our institutional review board (Samsung Medical Center, Seoul, Korea, (approval #2013-09-097)) approved this retrospective study and the requirement for informed consent was waived.

Details on study population, pulmonary function test, and imaging are provided in the File S1.

### Study sample

**Figure 1:**
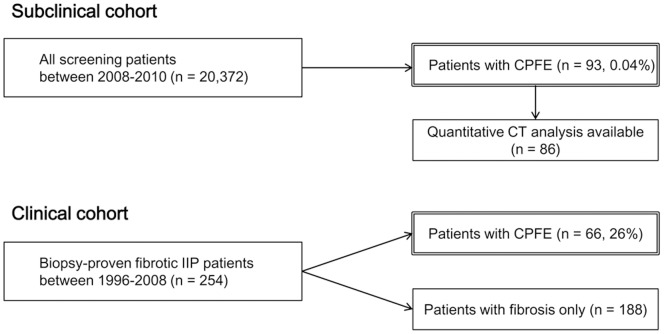
Flow chart of study design and patient population. CPFE = combined pulmonary fibrosis and emphysema; IIP = idiopathic interstitial pneumonia.

For the biopsy-proven CPFE cohort, we included a total of 254 consecutive patients seen from 1996 to 2008 at Samsung Medical Center who had pathologically confirmed fibrotic IIP on surgical lung biopsy. Cases of combined fibrotic IIP and emphysema were selected by two radiologists. The remaining 188 fibrotic IIP patients without emphysema were categorized into the fibrosis only cohort (Figure 1).

A separate CPFE cohort at the subclinical level was enrolled from the screening sample. At first, we acquired our patient data from 20,372 individuals who underwent chest CT for lung cancer screening or metastasis work-up and were found to have extrathoracic malignancy between June 2008 and May 2010. The same two radiologists reviewed all CT images, and 93 CPFE patients were identified. Seven of the 93 patients were excluded from this study due to difficulties interpreting CT images due to concurrent acute illness (n = 4) and poor imaging resolution (n = 3).

### Imaging and Interpretation

Another three independent chest radiologists analyzed the CT images [12]. The observers made subjective assessments of the overall extent of fibrosis-related lung parenchymal abnormalities. A score, which was estimated to the nearest 5% of parenchymal involvement compared to whole lung volume (100%), was assigned to each parenchymal abnormality. The total FS was calculated by adding the scores of each lung parenchymal abnormality.

The three observers reached the most likely diagnosis based on radiographic classification of the screening cohort [13]. They also evaluated the distribution of emphysema.

To assess EI, we used in-house computerized software [14]. Using volumetric CT, the volume fraction of the lung below −950 HU was calculated automatically and was defined as the EI [15].

### Derivation of Imaging-driven Indices

We derived five CT indices as follows: Fibrosis score (FS), emphysema index (EI), CPFE score, which combined FS and EI (FS+EI), fibrosis-weighted CPFE score, which combined doubled FS and EI (FS X 2+ EI), and emphysema-weighted CPFE score, which combined FS and doubled EI (FS+EI X 2).

### Statistical Analysis

Inter-reviewer agreement among the three radiologists was reported using an intra-class correlation coefficient for total fibrosis score. Patient demographics, as well as clinical and radiographic data, were compared across cohorts using the unpaired *t*-test for continuous variables, the Fisher’s exact test for categorical variables, and the log-rank test for survival curves. A *p*-value <0.05 was considered significant in all cases. Predictors of time to death were determined for patients using Cox proportional hazards analysis. Variables associated with time to death based on unadjusted analysis were considered for inclusion in the multivariate model. Statistical analyses were conducted using SPSS for Windows, version 15.0.

## Results

### Study sample and outcomes

Criteria for the biopsy-proven CPFE cohort were met in 66 of 254 fibrotic IIP patients, representing 26.0% of the overall cohort. In the subclinical setting, CPFE prevalence was 0.04% (93/20,372) (Figure 1). Baseline clinical and CT features of subclinical and biopsy-proven CPFE cohorts are compared in Table 1. Significant differences were observed in age, sex, and smoking history. Patients with subclinical CPFE were significantly older than those with biopsy-proven CPFE and were primarily male. A larger percentage of subclinical CPFE patients had a significant smoking history compared to biopsy-proven CPFE patients. Regarding PFT results, FVC was relatively more preserved in the subclinical CPFE cohort than in the biopsy-proven CPFE cohort (*p*<0.01). DLco results were available for 60 of 66 patients (91%) among the biopsy-proven CPFE cohort, and 42 of 86 patients (49%) in the subclinical CPFE cohort. DLco was lower in the subclinical CPFE cohort than in the biopsy-proven CPFE cohort. On CT, the subclinical CPFE cohort had less severe fibrosis (*p*<0.01). As for the subtypes of interstitial lung disease identified in the subclinical CPFE cohort, respiratory bronchiolitis (RB), RB-ILD, or desquamative interstitial pneumonia (DIP) patterns were identified in 12% of patients (*p*<0.01). No significant differences were noted in FEV1, emphysema distribution or EI between the biopsy-proven and subclinical CPFE cohorts.

**Table 1:** Baseline subject characteristics of two cohorts with combined pulmonary fibrosis and emphysema.

	Subclinical CPFE	Biopsy-proven CPFE	*P*-value
	n = 86	n = 66	
Age (y)	68.7±8.1 (41–87)	58.5±8.7 (44–72)	**<0.01**
Men (%)	85 (99)	50 (76)	**<0.01**
Ever smoker (%)	71 (83)	43 (65)	**<0.01**
Pulmonary function			
FEV_1_ (% predicted)	98.0±18.4	94.8±18.1	0.07
FVC (% predicted)	98.3±16.1	86.1±18.3	**<0.01**
DLco (% predicted)	69.6±21.4	78.0±20.1	**0.04**
CT features			
Subtypes of Infiltrative Lung Disease			**<0.01**
UIP	47 (55)	53 (80)	
NSIP	20 (23)	13 (20)	
RB-ILD or DIP	10 (12)		
Unclassifiable	9 (10)		
Emphysema distribution			0.12
Upper predominance	53 (62)	48 (73)	
Lower predominance	2 (2)	2 (3)	
Diffuse distribution	31 (36)	16 (24)	
Fibrosis score (range)	10.4±8.3 (5–45)	19.3±14.2 (5–75)	**<0.01**
Emphysema index (range)	11.3±10.4 (1.1–42.9)	9.1±7.1 (3.0–40.1)	0.18

Definitions of abbreviations: IIP = idiopathic interstitial pneumonia; FEV1 = forced expiratory volume in 1 second; FVC = forced vital capacity; DLco = diffusing capacity for carbon monoxide; UIP = usual interstitial pneumonia; NSIP = nonspecific interstitial pneumonia; RB-ILD = respiratory bronchiolitis-associated interstitial lung disease; DIP = desquamative interstitial pneumonia.

**Figure 2:**
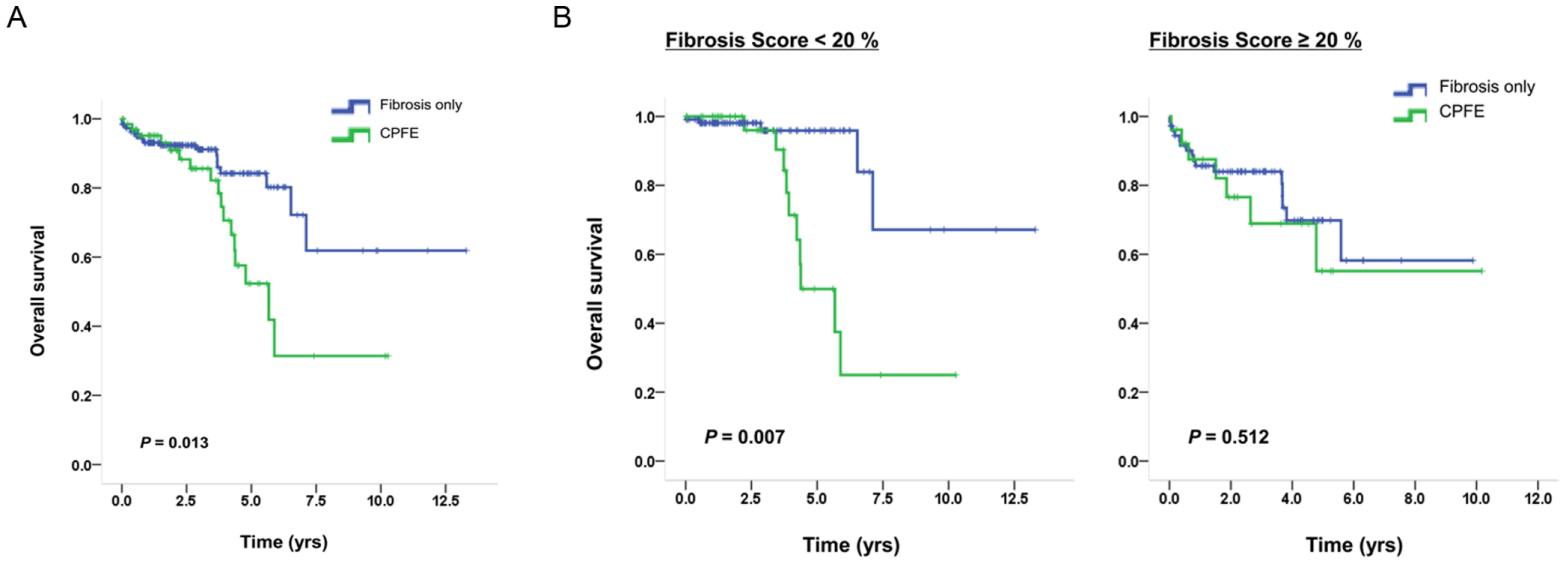
Kaplan-Meier survival curves of fibrotic IIP patients. (A) Stratified by CPFE vs. fibrosis only. (B) Stratified by CPFE vs. fibrosis only, separately with a cut-off value of 20% FS.

Regarding patient outcomes, median survival was 6.0 years and the 5-year survival rate was 34.8% in patients with CPFE, whereas it was 10.0 years and 60.9%, respectively, in fibrotic IIP patients without emphysema (*p* = 0.013). However, the severity of fibrosis did not differ significantly between the two cohorts (mean ± SD of FS; 19.3±14.2 and 19.5±12.6, respectively, *p* = 0.925) (Figure 2A). When Kaplan-Meier survival curves of fibrotic IIP patients were subgrouped with a cut-off value of 20% FS, there were significant survival differences in the subgroup with FS <20% (*p* = 0.007), but those difference were not seen in the subgroup with FS ≥20% (*p* = 0.512) (Figure 2B). The threshold of FS was chosen based on the trend of Kaplan-Meier survival curves in each 5%-class of FS (Figure S1).

The intra-class correlation coefficient for FS among three radiologists was 0.81 (95% confidence interval, 0.76–0.85).

### Prediction of Survival in the Biopsy-Proven CPFE Cohort

In patients with biopsy-proven CPFE, on univariate analysis, FVC and CPI were associated with greater risk of mortality as compared to similar degrees of dysfunction in FEV1 and DLco. Regarding CT features, the diagnosis of usual interstitial pneumonia (UIP) and fibrosis-weighted CT index were associated with higher mortality risk as compared to other CT-driven indices. On multivariate analysis, only FVC and fibrosis-weighted CT index were associated with a greater risk of mortality (Table 2).

**Table 2:** Univariate and multivariate hazard ratios for mortality in a biopsy-proven CPFE cohort.

Univariate variable	Hazard ratio	95% Confidence interval	*P*-value
Age	1.06	0.99–1.14	0.12
Men	0.41	0.12–1.44	0.17
Ever smoker	1.80	0.81–3.45	0.13
Pulmonary function			
FEV_1_ (% predicted)*	1.01	0.98–1.04	0.49
FVC (% predicted)*	1.07	1.00–1.14	**0.04**
DLco (% predicted)*	1.18	0.99–1.40	0.07
CPI	1.30	1.01–1.70	**0.03**
CT features			
UIP diagnosis	2.91	1.05–8.01	**0.02**
FS	1.05	0.98–1.12	0.16
EI	1.04	0.93–1.16	0.49
FS+EI	1.02	0.99–1.04	0.07
Fibrosis-weighted CT index	1.01	1.00–1.02	**0.04**
Emphysema-weighted CT index	1.02	0.99–1.04	0.11
**Multivariate variable**	**Hazard ratio**	**95% Confidence interval**	***P*** **-value**
FVC (% predicted)*	1.30	1.02–2.15	**0.04**
CPI	1.01	0.96–1.06	0.76
UIP diagnosis	0.74	0.12–4.69	0.75
Fibrosis-weighted CT index	1.82	1.47–7.04	**0.03**

Definitions of abbreviations: FEV1 = forced expiratory volume in 1 second; FVC = forced vital capacity; DLco = diffusing capacity for carbon monoxide; CPI = composite physiologic index; UIP = usual interstitial pneumonia; FS = fibrosis score; EI = emphysema index.

*Hazard ratios reported for 10-unit change.

### Prediction of Survival in the Subclinical CPFE Cohort

In patients with subclinical CPFE, a positive smoking history and fibrosis-weighted CT index were associated with greater mortality on univariate analysis. Of these factors, only fibrosis-weighted CT index was associated with mortality on multivariate analysis (Table 3).

**Table 3:** Univariate and multivariate hazard ratios for mortality in a subclinical CPFE cohort.

Univariate variable	Hazard ratio	95% Confidence interval	*P*-value
Age	3.19	0.51–20.0	0.21
Men	2.78	0.00–9.21	0.75
Ever smoker	2.87	1.65–10.03	**0.03**
Pulmonary function			
FEV_1_ (% predicted)*	1.81	0.57–5.81	0.32
FVC (% predicted)*	0.24	0.04–1.42	0.11
DLco (% predicted)*	0.40	0.12–1.30	0.13
CPI	0.11	0.01–1.22	0.07
CT features			
UIP diagnosis	8.97	0.00–29.01	0.73
FS	2.45	0.36–13.74	0.36
EI	1.96	0.09–40.62	0.66
FS+EI	1.06	0.97–1.15	0.16
Fibrosis-weighted CT index	2.45	1.09–5.36	**0.04**
Emphysema-weighted CT index	0.97	0.91–1.03	0.28
**Multivariate variable**	**Hazard ratio**	**95% Confidence interval**	***P*** **-value**
Ever smoker	3.03	0.94–9.78	0.06
Fibrosis-weighted CT index	1.01	1.00–1.03	**0.04**

Definitions of abbreviations: FEV1 = forced expiratory volume in 1 second; FVC = forced vital capacity; DLco = diffusing capacity for carbon monoxide; CPI = composite physiologic index; UIP = usual interstitial pneumonia; FS = fibrosis score; EI = emphysema index.

*Hazard ratios reported for 10-unit change.

## Discussion

Patients with IPF and emphysema are usually males with heavy cigarette-smoking histories who experience severe dyspnea on exertion with relatively conserved lung volumes despite disproportionate impairment of gas exchange [2], [16]. Emphysema affects baseline PFTs by increasing lung volumes and decreasing DL_CO_ and FEV1/FVC, as well as altering changes in these values over time. Ultimately, this influences the assessment of disease severity at baseline and over time [2], [16]. The inconsistent findings pertaining to CPFE impact on survival are a problem in the existing literature [17]–[22].

Ryerson [17] recently reported prevalence rates, clinical features, and CPFE prognosis in IPF. An analysis of 29 patients with CPFE among 265 IPF patients showed that there was no significant difference in mortality between CPFE and fibrosis only cohorts (hazard ratio 1.14, 95% confidence interval 0.61–2.13, *p = *0.69). These results seem to contradict the results of the present study and those of several other related studies [23]. We believe that these inconsistent results stem from the heterogeneous study populations involved in these studies, as well as the fact that predominant conclusions were often based on subgroup analyses [17], [23]. Ryerson [17] defined CPFE as ≥10% emphysema on HRCT. A threshold of 10% has previously been used to define CPFE with excellent inter-rater reliability (kappa 0.89) [18], and the Global Initiative for Chronic Obstructive Lung Disease (GOLD) suggested that this level of emphysema is expected to have symptomatic and physiologic consequences [24]. Consequently, the prevalence of CPFE detected by Ryerson [17] as significantly lower than in previous studies (8% vs. 18.8 to 50.9%) [23]. More importantly, in the study population, patients with CPFE had less HRCT fibrosis compared to non-CPFE patients, and the similar mortality rates seen in patients with CPFE and IPF without emphysema is a reflects that the mortality risk factors between CPFE (poor oxygenation and greater pulmonary hypertension) and IPF without emphysema (greater degrees of fibrosis) were approximately balanced. On the other hand, in the present study, CPFE was associated with significantly higher mortality than fibrosis only (*p* = 0.013), even though the severity of fibrosis did not differ significantly between the two groups (*p* = 0.925).

We additionally compared two groups stratified by a FS of 20% on CT, and found that the difference in mortality was relevant only for FSs <20%. We also found that individual PFT measures varied in terms of predicting patient survival in both cohorts. The interaction between emphysema and fibrosis was very complicated. Effects on survival may differ according to degrees of fibrosis or emphysema, making the clinical relevance of these results all the more difficult to interpret. Recently, Schmidt *et al.* compared CPFE and IPF without emphysema [11] and found that individual measures of pulmonary function vary in their abilities to predict mortality based on the quantity of emphysema, a finding that could be interpreted in the same context.

We found that a fibrosis-weighted CT index was a significant predictor of prognosis among a variety of predictive variables in patients with both biopsy-proven and subclinical CPFE. These findings may be partially explained by the effect of emphysema in a subgroup with less severe fibrosis (<20%). We believe that our study is the first trial to quantitatively measure EI and FS together in CPFE patients, and to assess and evaluate the prognostic significance of these two CT-based indices/scores. Over the years, there have been significant advancements in HRCT technology, and HRCT has become the standard approach to radiographic evaluations of pulmonary fibrosis [9]. The use of quantitative HRCT information may refine such analyses and expand on these findings. EI is a value that can be easily acquired and is correlated well with physiologic results or autopsy specimens [25]. Furthermore, FS is a well-known radiographic predictor of IPF [2]. We applied quantitative CT analysis reflecting entire lung data as compared to the subjective visual assessments or limited image analyses used in previous studies [11], [17]. Therefore, we provide more accurate and localized evaluations of disease severity than previous studies.

Our data highlight that the use of a fibrosis-weighted CT index is a better surrogate for PFTs or CPI in predicting subsequent mortality in patients with both biopsy-proven and subclinical CPFE. Of course, our DLco data may not be conclusive, given that data were not available for all patients. However, our results were similar to those reported in a recent study conducted by Schmidt *et al.* [11], in which DLco was not a significant predictor of mortality in emphysema patients. In that study, there was a possibility of selection bias, as more severely ill patients may be unable to perform DLco tests. In contrast to such limitations, CT is an easily accessible imaging tool regardless of symptom severity.

Our patients with fibrotic IIPs had milder radiologic fibrosis than previously reported IPF patients, a finding that may help explain the higher survival rates among this cohort than in previous studies. We selectively included patients who were scheduled to undergo surgical lung biopsy for IIP diagnosis. Patients with typical UIP on imaging and who were not candidates for biopsy were excluded. However, we focused more on subclinical or early manifestations of IIP since clinical and research settings indicate that HRCT facilitates the detection of ILD in asymptomatic and undiagnosed individuals [26]. We found a substantial compounding effect of emphysema on survival in fibrotic IIP patients and less severe fibrosis. This reflects the necessity of longitudinal and large-cohort studies of patients with subclinical ILD. In the same context, we applied our analyses to two different cohorts, a cancer screening cohort (subclinical cohort) and another fibrotic IIP cohort (biopsy-proven cohort). Results for these two cohorts were compared in order to identify the prevalence of CPFE with reference to definite IPF as well as the subclinical stage prior to definite IPF. In the two cohorts, FVC and DLco worked differently; this indicates the importance of assessing CPFE at the subclinical level. The detection of subclinical fibrosis is increasing due to increased rates of lung cancer screening *via* CT.

The current study had several limitations. First, our study was retrospectively designed at a single tertiary center. Therefore, some selection bias may have affected our findings. Second, the results of physiologic tests were unavailable, especially for the subclinical cohort. Third, we did not consider other prognostic factors such as pulmonary hypertension; we evaluated only physiologic and CT features as predictors of patient survival.

In conclusion, recognition and stratification of CPFE using quantitative CT analysis may be an important prognostic tool. In univariate and multivariate analyses, prognostic predictive factors for survival may vary according to the severity of fibrosis and patient characteristics, including whether the study cohort consists of patients with biopsy-proven or a subclinical CPFE.

## Supporting Information

Figure S1 **Kaplan-Meier survival curves of fibrotic IIP patients in each 5%-class of FS, stratified by CPFE vs. fibrosis only (online only).**
(TIF)

File S1 (DOCX)
