# Supplementary figures and images for: The Value of CT for Disease Detection and Prognosis Determination in Combined Pulmonary Fibrosis and Emphysema (CPFE)
Source: PLoS One. 2014 Sep 9;9(9):e107476. doi: 10.1371/journal.pone.0107476 (PMC4159339; doi:10.1371/journal.pone.0107476)

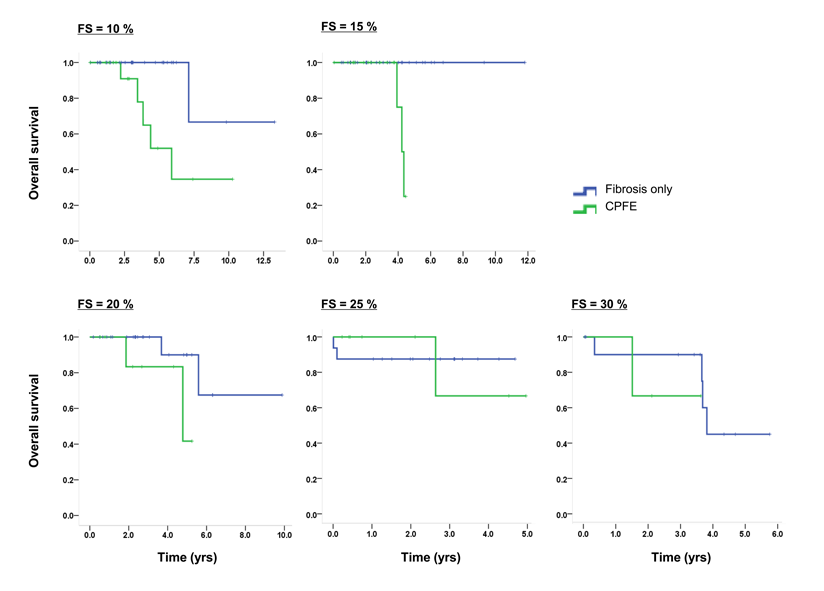

Supplement: Figure S1 — Kaplan-Meier survival curves of fibrotic IIP patients in each 5%-class of FS, stratified by CPFE vs. fibrosis only (online only). (TIF) [file pone.0107476.s001.tif]
