# Supplementary material for: The Value of CT for Disease Detection and Prognosis Determination in Combined Pulmonary Fibrosis and Emphysema (CPFE)
Source: PLoS One. 2014 Sep 9;9(9):e107476. doi: 10.1371/journal.pone.0107476 (PMC4159339; doi:10.1371/journal.pone.0107476)
Supplement: File S1 — (DOCX) [file pone.0107476.s002.docx]

**METHODS**
This retrospective study, conducted at a single tertiary-care center, was approved by our institutional review board (approval #2013-09-097). Informed consent was waived for the use of patient medical data.
 ***Study population***
For the clinical CPFE cohort, we included a total of 254 consecutive patients who had pathologically confirmed fibrotic IIP on surgical lung biopsy and were seen from 1996 to 2008 at Samsung Medical center. All patients had both HRCT and PFT data obtained within 6 months of surgical lung biopsy. Cases of fibrotic IIP combined with emphysema were selected by two radiologists, both of whom had interstitial lung disease (ILD) HRCT interpretation experience of more than 5 years, and one of whom was the guarantor of this entire study. We included a total of 66 CPFE patients in the fibrotic IIP cohort. The remaining 188 fibrotic IIP patients without emphysema were categorized into the fibrosis only group (Figure 1).
A separate CPFE population at the subclinical level was enrolled from the screening cohort. We acquired patient data from all 20,372 individuals who underwent chest CT for the purpose of lung cancer screening or metastasis work-up found to have extrathoracic malignancy between June 2008 and May 2010. The same two radiologists reviewed all CT images, and 93 CPFE patients were identified (Figure 1). Seven of the 93 patients were excluded from this study because of the difficulty in CT image interpretation due to concurrent acute illness (n = 4) and poor imaging resolution (n = 3). Finally, among the screening cohort, 86 patients were classified as the subclinical level and were included in CT analysis of CPFE (Figure 1).
  ***Pulmonary Function Tests and Patient Survival***A chest physician (with 17 years of ILD management experience) reviewed clinical data through patient hospital records. Data included age, sex, smoking history (pack-years), and PFT results. FVC, FEV1, FEV1/ FVC, and DLco were determined based on pulmonary function testing and were expressed as the percentage of predicted value based on height, age, sex and ethnicity. Survival period and cause of death were collected from medical records. Patient families were directly contacted and the national health registry was accessed.  ***Imaging and Interpretation***HRCT scans of all patients were obtained at the end inspiration in the supine position using a variety of scanners without IV contrast material. The protocols consisted of 1- to 2-mm collimation sections reconstructed with a high-spatial-frequency algorithm at 1- or 2-cm intervals. All images were viewed on a workstation, at window settings appropriate for viewing lung parenchyma (window level, −600 or −700 Hounsfield units [HU]; window width, 1,500 or 1,750 HU) and mediastinum (window level, 40-50 HU; window width, 250-400 HU) [[1](#_ENREF_1)].
Three independent chest radiologists (with 20, 13, and 11 years of HRCT interpretation experience, respectively) blinded to clinical history (including PFT, or pathologic data) analyzed the CT images. The observers made subjective assessments of the overall extent of fibrosis-related lung parenchymal abnormalities, as well as the extent of reticulation and honeycombing. All HRCT images were observed from the thoracic inlet to the lung base. A score, which was estimated to the nearest 5% of parenchymal involvement, compared whole lung volume (100%) and was assigned to each parenchymal abnormality. The total FS was calculated by adding the scores of each lung parenchymal abnormality. Because three reviewers took part in the reading session, the final score for the total extent and for the extent of each parenchymal abnormality was calculated by averaging the scores from the three reviewers.
After scoring the CT scans, three observers reached a most likely diagnosis based on criteria established by the American Thoracic Society and the European Respiratory Society relating to radiographic classifications of screening cohorts [[2](#_ENREF_2)]. They also visually evaluated the distribution of emphysema with axial and coronal images, including upper and lower predominance and diffuse distributions. The predominant craniocaudal distribution of emphysema was classified as predominantly in the upper lungs, lower lungs, or diffusely throughout. Inconsistencies between individual reviewers were resolved by consensus.
To assess EI, we used an in-house computerized software [[3](#_ENREF_3)]. Based on volumetric CT, the whole lung and airway was extracted automatically by 3D regional growing methods with the threshold of - 400 HU. Secondly, semiautomatic removal of the airway was performed using the following methods. Simple thresholding below - 950 HU was performed by using 3D regional growing methods with the seed point at the trachea. Then, 3D morphologic dilation was performed on the segmented airway. The segmented lung was subtracted by the segmented airway. The volume fraction of the lung below - 950 HU was calculated automatically and was deﬁned as EI [[4](#_ENREF_4)].

**References**

1. Lee HY, Seo JB, Steele MP, Schwarz MI, Brown KK, et al. (2012) High-resolution CT scan findings in familial interstitial pneumonia do not conform to those of idiopathic interstitial pneumonia. Chest 142: 1577-1583.

2. Travis WD, Costabel U, Hansell DM, King TE, Jr., Lynch DA, et al. (2013) An official american thoracic society/european respiratory society statement: update of the international multidisciplinary classification of the idiopathic interstitial pneumonias. Am J Respir Crit Care Med 188: 733-748.

3. Park YS, Seo JB, Kim N, Chae EJ, Oh YM, et al. (2008) Texture-based quantification of pulmonary emphysema on high-resolution computed tomography: comparison with density-based quantification and correlation with pulmonary function test. Invest Radiol 43: 395-402.

4. Chae EJ, Seo JB, Song JW, Kim N, Park BW, et al. (2010) Slope of emphysema index: an objective descriptor of regional heterogeneity of emphysema and an independent determinant of pulmonary function. AJR Am J Roentgenol 194: W248-255.
